# Prediction accuracy measurements as a fitness function for software effort estimation

**DOI:** 10.1186/s40064-015-1555-9

**Published:** 2015-12-15

**Authors:** Tomas Urbanek, Zdenka Prokopova, Radek Silhavy, Veronika Vesela

**Affiliations:** Department of Computer and Comunication systems, Tomas Bata University in Zlin, Nad Stranemi 4511, Zlin, Czech Republic

**Keywords:** Effort estimation, Software engineering, Use case points, Analytical programming, Differential evolution, Prediction accuracy measures

## Abstract

This paper evaluates the usage of analytical programming and different fitness functions for software effort estimation. Analytical programming and differential evolution generate regression functions. These functions are evaluated by the fitness function which is part of differential evolution. The differential evolution requires a proper fitness function for effective optimization. The problem is in proper selection of the fitness function. Analytical programming and different fitness functions were tested to assess insight to this problem. Mean magnitude of relative error, prediction 25 %, mean squared error (MSE) and other metrics were as possible candidates for proper fitness function. The experimental results shows that means squared error performs best and therefore is recommended as a fitness function. Moreover, this work shows that analytical programming method is viable method for calibrating use case points method. All results were evaluated by standard approach: visual inspection and statistical significance.

## Background

Effort estimation is defined as the activity of predicting the amount of effort required to complete a development of software project (Keung 2008). It is necessary to predict the effort estimation in the early stages of the software development cycle. In the best case, estimates should be calculated after a requirement analysis (Karner 1993).

Effort estimation methods can be divided into two major groups algorithmic methods and non-algorithmic methods. Algorithmic methods carries mathematical formula, which is regression model of historical data. The most famous methods are COCOMO (Boehm 1984), FP (Atkinson and Shepperd 1994) and UCP (Karner 1993). But there is a lot of algorithmic methods. To the second category belong methods like expert judgement and analogy based methods. The most famous methods is Delphi (Rowe and Wright 1999).

The use of artificial intelligence may be a promising way to improve the accuracy of effort estimations. Accurate and consistent estimates are crucial in software project management. These estimates are used for the effective planning, monitoring and controlling of a software development cycle. Project managers may use these estimates to arrive at better management decisions. Software engineering is a complicated process because there are a lot of factors—for example, the size of the development team, the actual requirements, the programming language used, as well as other factors. These factors may have a considerably impact on the accuracy of the effort estimation process.

In this research study, the analytical programming method was used to improve the use case points method. The Use Case Points method is widely used for effort estimation in software engineering. The main benefit of this method is that it provides effort estimates at a relatively early stage in the software development cycle. Nevertheless, this method is fully dependent on the human factor since the project manager has to estimate the project parameters and set the weights. There is a low probability that two project managers will perform these estimates exactly alike. Therefore, this research uses artificial intelligence to account for this dependency on the human factor. At the same time, this method is based on straightforward computation and allows a wide range of calibration—which can be achieved by setting the weights. The combination of analytical programming and the use case points method is used to derive early and more accurate effort estimation results. Analytical programming—as a symbolic regression technique, could be used to create a new model for the use case points method. An appropriate fitness function is vital for this task (Harman and Jones 2001). The fitness function evaluates solutions and decides whether the solution is acceptable—or not, for further processing. There are a large number of prediction accuracy measurement methods for assessing the accuracy of a predictive model. Thus, in this field, one of the main obstacles is to report the accuracy correctly. MMRE or Pred(25) are mainly used for the evaluation of the statistical properties of predictive models in the software engineering field. Currently, the MMRE method is being criticised by some experts in this field—e.g., in Myrtveit et al. (2003), Shepperd et al. (2000) or Kitchenham et al. (2001); however, the method is de-facto considered as a standard for reporting the suitability of a proposed model. In this study, prediction accuracy measurements will be used as fitness functions for the analytical programming.

The Sect. “Related work” of this paper summarise the related work in this field. Section “Problem statement” present the research questions for this work. Section “Experiment planning” describes the methodology used for this study. Section “Results” is devoted to the results of this work. In the next section you can see the limitations of this study. And finally, Sect. “Discussion” present discussion and conclude this paper.

### The use case points method: short description

This effort estimation method was presented in 1993 by Karner (1993). It is based on a similar principle to the function point method. Project managers have to estimate the project parameters to four tables. These tables are as follows:

- Unadjusted use case weight (UUCW)
- Unadjusted actor weight (UAW)
- Technical complexity factor (TCF)
- Environmental complexity factor (ECF)

#### Unadjusted use case weight

The UCP method includes three categories for use case classification, which concern the use case complexity of the developed system. All the categories with weights are presented in Table 1. The influence of actor classification (UCW) are assessed by summing the number of use case with corresponding weights, see the Eq. 1.

**Table 1:** UCP table for estimation unadjusted use case weight

Use case classification	No. of transactions	Weight
Simple	1–3 transactions	5
Average	4–7 transactions	10
Complex	8 or more transactions	15

1 $$\begin{aligned} UUCW = \sum \limits _{i\in C}uClassification(c)*uWeight(c), \end{aligned}$$

where $C\in \{simple,average,complex\}$ as can be seen in Table 1.

#### Unadjusted actor weight

The UCP method includes three categories for actor classification, which concern the actor complexity of the developed system. All the categories with weights are presented in Table 2. The influence of actor classification (UAW) are assessed by summing the number of actors with corresponding weights, see the Eq. 2.

**Table 2:** UCP table for actor classification

Actor classification	Weight
Simple	1
Average	2
Complex	3

2 $$\begin{aligned} UAW = \sum \limits _{i\in C}aClassification(c)*aWeight(c), \end{aligned}$$

where $C\in \{simple,average,complex\}$ as can be seen in Table 2.

#### Technical complexity factor

The UCP method includes 13 technical factors, which concern the technical complexity of the developed system. All the technical factors are presented in Table 3. The influence of technical complexity factors (TCF) are assessed by assigning a value from 0 to 5 to each of them. This value is multiplied by a weight of a factor and totaled, see the Eq. 3.

**Table 3:** UCP table for technical factor specification

Factor	Description	Weight
T1	Distributed system	2.0
T2	Response time/performance objectives	1.0
T3	End-user efficiency	1.0
T4	Internal processing complexity	1.0
T5	Code re-usability	1.0
T6	Easy to install	0.5
T7	Easy to use	0.5
T8	Portability to other platforms	2.0
T9	System maintenance	1.0
T10	Concurrent/parallel processing	1.0
T11	Security features	1.0
T12	Access for third parties	1.0
T13	End user training	1.0

3 $$\begin{aligned} TCF = 0.6 + \left( 0.01 * \sum \limits _{i=1}^{13}Value_i*Weight_i\right) \end{aligned}$$

#### Environmental complexity factor

The UCP method includes 8 environmental factors, which concern the environmental complexity of the developed system. All the environmental factors are presented in Table 4. The influence of environmental complexity factors (ECF) are assessed by assigning a value from 0 to 5 to each of them. This value is multiplied by a weight of a factor and totaled, see the Eq. 4.

**Table 4:** UCP table for environmental factor specification

Factor	Description	Weight
E1	Familiarity with development process used	1.5
E2	Application experience	0.5
E3	Object-oriented experience of team	1.0
E4	Lead analyst capability	0.5
E5	Motivation of the team	1.0
E6	Stability of requirements	2.0
E7	Part-time staff	−1.0
E8	Difficult programming language	−1.0

4 $$\begin{aligned} ECF = 1.4 + \left( -0.03 * \sum \limits _{i=1}^{8}Value_i*Weight_i\right) \end{aligned}$$

#### Final equations

The Eq. 5, is used for the calculation of the number of use case points. This number of use case points then has to be multiplied by productivity factor in order to obtain the effort estimation result, i.e., Eq. 6. This productivity factor was chosen by Karner (1993), and was set to default value 20 h per UCP. The calibration of use case points will be performed by replacing the Karner’s equation for new model. This new model will be built by analytical programming method.

5 $$\begin{aligned} UCP = (UUCW + UAW) * TCF * ECF \end{aligned}$$

6 $$\begin{aligned} EE = UCP * PF \end{aligned}$$

### Optimization tools

In this research, we use analytical programming method with differential evolution algorithm to calibrate use case points method.

#### Analytical programming

Analytical programming (AP), is a symbolic regression method. The core of analytical programming is a set of functions and operands. These mathematical objects are used for the synthesis of a new function. Every function in the analytical programming set core has its own varying number of parameters. The functions are sorted according to these parameters into general function sets (GFS). For example, $GFS_{1par}$ contains functions that have only 1 parameter—e.g., *sin*(), *cos*(), or other functions. AP must be used with any evolutionary algorithm that consists of a population of individuals for its run (Zelinka et al. 2011; Oplatkova et al. 2013). In this paper, Differential evolution (DE) is used as an analytical programming evolutionary algorithm.

**Fig. 1:**
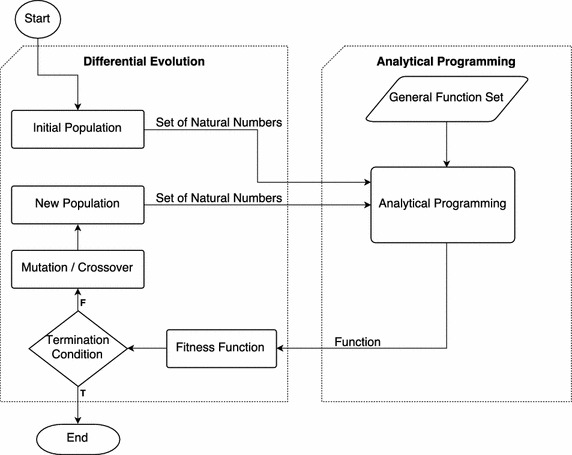
Scheme of analytical programming with differential evolution algorithm

The function of analytical programming can be seen in Fig. 1. In this case, the evolutionary algorithm is a differential evolution. The initial population is generated using differential evolution. This population, which must consist of natural numbers, is used for analytical programming purposes. The analytical programming then constructs the function on the basis of this population. This function is evaluated by its fitness function. If the termination condition is met, then the algorithm ends. If the condition is not met, then differential evolution creates a new population through the mutation and recombination processes. The whole process continues with the new population. At the end of the analytical programming process, it is assumed that one has a function that is the optimal solution for the given task.

#### Differential evolution

Differential evolution is an optimisation algorithm introduced by Storn and Price (1995). This optimisation method is an evolutionary algorithm based on population, mutation and recombination. Differential evolution is easy to implement and has only four parameters which need to be set. The parameters are: generations, NP, F and Cr. The generations parameter determines the number of generations; the NP parameter is the population size; the F parameter is the weighting factor; and the Cr parameter is the crossover probability (Storn 1996). In this research, the differential evolution is used as an analytical programming engine.

### The fitness function

The fitness function is a mathematical formula that assesses the appropriateness of the solution of a given task. The selection of the appropriate fitness function is one of the most important tasks in designing an evolutionary process (Harman and Jones 2001). In the case of this study, the prediction accuracy measurements are used as fitness functions. These measurements are commonly used for the evaluation of the predictive model. It is assumed that this use of predictive accuracy measurements allows one to determine the behaviour of different fitness functions. These knowledge will be important for future research.

## Related work

Some work has been done to enhance the effort estimation based on the use case points method. These enhancements cover the review and calibrating the productivity factor such as the work of Subriadi and Ningrum (2014). Another enhancement could be the construction investigation and simplification of the use case points method presented by Ochodek et al. (2011). The recent work of Silhavy et al. (2014) suggest a new approach “automatic complexity estimation based on requirements”, which is partly based on use case points method. Or using fuzzy inference system approach to improve accuracy of the use case points method (Nassif et al. 2011). Surveys such as that conducted by Kitchenham et al. (2001), have shown that MMRE measures the spread (i.e. standard deviation). Therefore, this measurement is not suitable for accuracy predictions. The same study also showed that Pred(25) is a measurement of Kurtosis. Thus far, several studies such as Burgess et al. (2001), Chavoya et al. (2013) and Chavoya et al. (2013) have tested the efficiency of using the genetic programming method for more accurate effort estimation. In 2010, Ferrucci et al. (2010) published a paper in which they used a similar principle to assess accuracy by using different fitness functions. The authors used genetic programming and the function point method for their research. Genetic programming can suffer on bloat effect and constant resolving. In this research study on the other hand, a combination of analytical programming and the use case points method were used. There is no bloat effect in analytical programming because model is built by giving the length of the model. The problem of constant resolving can be solve by meta-evolution or non-linear fitting, e.g., Levenberg-Marquardt algorithm.

## Problem statement

The overall research question to be answered within the study is whether there is a possibility to outperformed the Karner’s equation by analytical programming method and is there a fitness function which outperforms the other fitness functions. This section presents the design of the research questions we carried out to get an insight in the use of analytical programming for effort estimation. The research questions of our study can be outlined as follows:

- *RQ-1* Comparing the estimates achieved by applying analytical programming with the estimates obtained by standard use case points method equation.
- *RQ-2* Analysing the impact of different fitness functions on the accuracy of the estimation models built with analytical programming.

The first research question (RQ-1) aims to get an insight on the estimation accuracy of analytical programming and understand the actual effectiveness of this technique with respect to the estimates by standard use case points method. For this reason, we first calibrate the UCP equation to produce the best estimates. Then, we try to outperformed this estimates by the method of analytical programming. The same process was carried out for standard calibration of UCP method. To address research question (RQ-2) we experimented with ten different fitness functions as reported and discussed in experiment planning section. To asses the performance of fitness function we used descriptive statistics and Wilcox signed rank test.

## Experiment planning

**Fig. 2:**
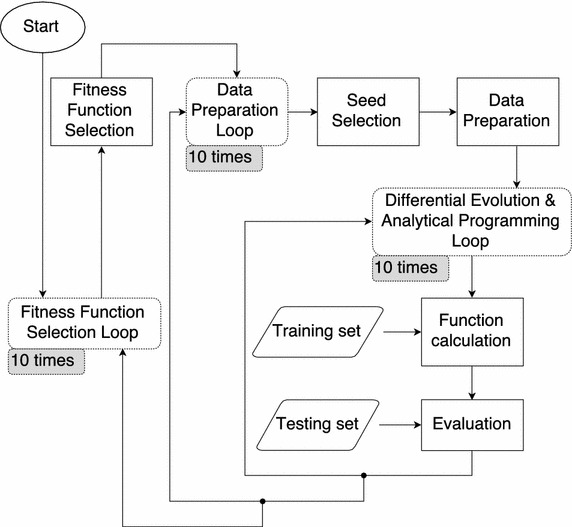
Diagram of proposed experiment

The proposed experiment can be seen in the Fig. 2. The process begins with a cycle that loops through the number of used fitness functions. In this case, there are ten fitness functions. Ten different seeds were used to assess the reliability of the proposed experiment. In the data preparation loop, the seed was used to split the dataset into to two distinct sets. The dataset was split into the ratio of 66  % (i.e., training set) and 33  % (i.e. testing set). The dataset is depicted in Table 5. Then, there is a third loop that runs 10 times. In this loop, the differential evolution process starts to generate an initial population. Analytical programming then uses this initial population to synthesise a new function. After that, the new function is evaluated by the one of the selected fitness functions. If the termination condition is met, one can assume that one has an optimal predictive model, and this model is then evaluated by the calculation of the least absolute deviation (LAD) on the testing set. Then, the results are saved to file for further analysis. It is necessary to note that 10 different seeds are used for every of 10 models, as well as one of the 10 fitness functions. Thus, we have a total of 10 $\times$ 10 $\times$ 10 solutions.

### Dataset

The data for this study was collected using document reviews. The use case points method dataset was obtained from Poznan University of Technology (Ochodek et al. 2011) and from Subriadi’s paper (Subriadi and Ningrum 2014).

**Table 5:** Data used for effort estimation

ID	UUCW	UAW	TCF	ECF	Actual effort (man/h)
1	195	12	0.780	0.780	3037
2	80	10	0.750	0.810	1917
3	75	6	0.900	1.050	1173
4	130	9	0.850	0.890	742
5	85	12	0.820	0.790	614
6	50	9	0.850	0.880	492
7	50	6	0.780	0.510	277
8	305	14	0.940	1.020	3593
9	85	12	1.030	0.800	1681
10	130	12	0.710	0.730	1344
11	80	9	1.050	0.950	1220
12	70	12	0.780	0.790	720
13	30	4	0.960	0.960	514
14	100	15	0.900	0.910	397
15	355	15	1.125	0.770	3684
16	145	18	1.080	0.770	1980
17	325	12	1.095	0.935	3950
18	90	6	1.085	1.085	1925
19	125	9	1.025	0.980	2175
20	120	9	1.115	0.995	2226
21	200	12	1.000	0.920	2640
22	175	9	0.950	0.920	2568
23	245	12	0.890	1.190	3042
24	140	6	0.965	0.755	1696

**Fig. 3:**
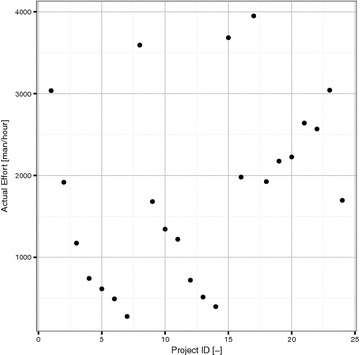
The distribution of actual efforts

Table 5 displays the use case points method data from 24 projects. Only the use case points method data with transitions were utilized in this paper in the case of the Poznan University of Technology dataset. There are 5 values for each software project: UUCW, UAW, TCF, ECF and actual effort. Software projects 1–14 are from Poznan University of Technology. The rest are from Subriadi’s paper. As can be seen Subriadi’s data are quite consistent in actual effort. The possible reason is that these projects are related to one context, respectively linked to the web development software projects. The distribution of actual effort of this dataset can be seen on Fig. 3.

**Table 6:** Set-up of analytical programming

Parameter	Value
Number of leafs	30
GFS-functions	Plus, subtract, divide, multiply, tan, sin, cos
GFS-constants	UUCW, UAW, TCF, ECF, K

Table 6 shows the analytical programming set-up. The number of leafs (functions built by analytical programming can be seen as trees) was set at 30, which can be recognized as a relatively high value. However, one needs to find the model that will be more accurate than the Karner’s model. There is no need to generate short and easily memorable model, but rather, model that will be more accurate.

**Table 7:** Set-up of differential evolution

Parameter	Value
NP	40
Generations	60
F	0.7
Cr	0.4

Table 7 shows the set-up of differential evolution. The best set-up of differential evolution is the subject of further research.

### Fitness functions

The new model built by the analytical programming method contains the following parameters: UUCW, UAW, TCF and ECF. There is no force applied to the analytical programming that the models built by the analytical programming method have to contain all of these parameters. Ten different fitness functions (i.e., prediction accuracy measurements) were applied in this research.

**Table 8:** Used prediction accuracy measures

ID	Name	Equations
1	Least absolute deviations (LAD)	$LAD=\sum \limits _{i=1}^{n}\left| y_i-\hat{y}_i\right|$
2	Mean absolute error (MAE)	$MAE=\frac{1}{n}\sum \limits _{i=1}^{n}\left| y_i-\hat{y}_i\right|$
3	Mean squared error (MSE)	$MSE=\frac{1}{n}\sum \limits _{i=1}^{n}\left( y_i-\hat{y}_i\right) ^2$
4	Root mean squared error (RMSE)	$RMSE=\sqrt{\frac{1}{n}\sum \limits _{i=1}^{n}\left( y_i-\hat{y}_i\right) ^2}$
5	Mean magnitude of relative error (MMRE)	$MMRE=\frac{1}{n}\sum \limits _{i=1}^{n}\frac{\left| y_i-\hat{y}_i\right| }{y_i}$
6	Median magnitude of relative error (MdMRE)	$MdMRE=median\left( \frac{1}{n}\sum \limits _{i=1}^{n}\frac{\left| y_i-\hat{y}_i\right| }{y_i}\right)$
7	MMRE relative to the estimate (MEMRE)	$MEMRE\,=\,\frac{1}{n}\sum \limits _{i=1}^{n}\frac{\left| y_i-\hat{y}_i\right| }{\hat{y}_i}$
8	MdMRE relative to the estimate (MdEMRE)	$MdEMRE\,=\,median\left( \frac{1}{n}\sum \limits _{i=1}^{n}\frac{\left| y_i-\hat{y}_i\right| }{\hat{y}_i}\right)$
9	R squared ($R^2$)	$R^2=1-\frac{\sum \limits _{i\,=\,1}^{n}\left( y_i-\hat{y}_i\right) ^2}{\sum \limits _{i=1}^{n}\left( y_i-\bar{y}\right) ^2}$
10	Prediction within 25 % Pred(25)	$Pred(25)=\frac{Number\;of\;projects,\;where\;(MRE\le 0.25)}{Number\;of\;projects}$

Table 8 shows the prediction accuracy measurements used. These equations were used for the learning algorithm. Standard accuracy measurements in the software engineering field—like MMRE or Pred(25) were chosen. Moreover, accuracy measurements used for general purposes—like the LAD or MSE methods were also chosen. For equations from 1 to 8; when the equation result is closer to zero, then the accuracy of the proposed model is higher. On the other hand, this condition does not apply for Eqs. 9 and 10—namely, the R squared ($R^2$) method and the prediction within 25  % Pred(25) method. The result of the $R^2$ method ranges from 0 to 1, and the accuracy of the proposed model is higher when $R^2$ is closer to 1. Likewise, the same conditions apply for Pred(25).

## Results

In this section, we present the result of our study. Exploratory statistical analysis and hypothesis testing were utilized to describe research results. All the calculations was performed on testing dataset, which consist of 8 randomly chosen data from dataset. To obtain the average error for one project one need to divide the error by value 8.

**Fig. 4:**
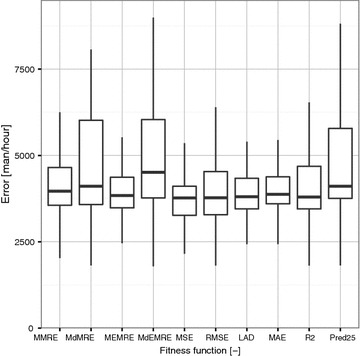
Statistics for each fitness function (one *box* is calculated from 100 equations and for testing dataset)

Figure 4 provide Statistics for each fitness function. As can be seen on this graph, nearly all fitness functions have a median value about 4000 man/h. On this figure could be also seen a considerably worse statistical properties for MdEMRE, Pred25 and MdMRE. As can be notice nearly all fitness functions have a minimum value about 2500 man/h. The exact values can be seen in Table 9.

**Table 9:** Summary statistics for each prediction accuracy measure

Fitness function	Values are calculated by least absolute deviation (LAD) (man/h)					
	Min.	1st Qu.	Median	Mean	3rd Qu.	Max.
MMRE	1264	3556	3966	14,366	4652	1,000,000
MdMRE	1815	3579	4109	4740	6019	12,975
MEMRE	1844	3483	3838	14,136	4368	1,000,000
MdEMRE	1790	3771	4514	15,481	6038	1,000,000
MSE	1813	3268	3769	3921	4106	9613
RMSE	1810	3284	3775	24,047	4533	1,000,000
LAD	1816	3454	3805	12,627	4337	475,584
MAE	1728	3599	3874	4094	4380	7842
$R^2$	1810	3452	3794	24,577	4687	1,000,000
Pred(25)	1815	3758	4107	24,774	5785	1,000,000

Table 9 provides the summary statistics for each fitness function. The minimum value of the minimum was calculated by MMRE, which is considerably lower then minim values of other fitness function. The most surprising aspect of the data is in the calculation of maximum value for MdMRE, MSE, LAD and MAE. These fitness functions does not reach the penalisation maximum. The penalisation maximum was set to 1,000,000 and in calculations for almost each equation was reached in about 1–2 %. The median value for every cost function is about 4000 man/h.

**Fig. 5:**
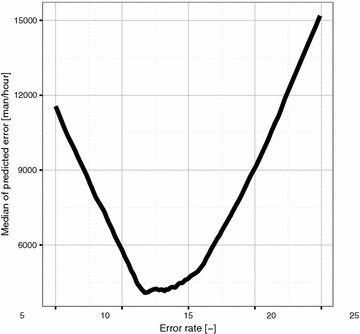
Median statistics of prediction error for standard UCP equation on testing dataset

Figure 5 shows the median of predicted error on testing data for Eq. 5. As can be seen the optimal productivity factor for testing dataset is between 11 and 14. The productivity factor value of 20, which is widely used, produce median error of 7469 man/h. Minimum value is 3227 man/h, if the error was set to 11.8. The median value of 3227 man/h was used as a value which need to be outperformed to have better results than from standard UCP Eq. 5.

### Optimal productivity factor

The optimal productivity factor was set according to Fig. 5. The minimum value is 3227 man/h, if the productivity factor was set to 11.8. The Wilcox signed rank test for one sample was used to determine which fitness function have a location shift lower than 3227 man/h. All calculation was performed on 95 % significance level.

**Table 10:** Hypothesis testing for optimal productivity factor

Fitness function	p value	NULL hypothesis
MMRE	9.29E$-$12	False
MdMRE	7.50E$-$14	False
MEMRE	2.98E$-$11	False
MdMRE	7.40E$-$16	False
MSE	5.26E$-$08	False
RMSE	9.56E$-$10	False
LAD	1.42E$-$10	False
MAE	4.24E$-$10	False
$R^2$	5.27E$-$11	False
Pred(25)	1.56E$-$14	False

Table 10 provides the results of Wilcox signed rank test for one sample. Every fitness function was tested on NULL hypothesis that this fitness function have lower true location than 3227 man/h. The value of “True” means that NULL hypothesis was accepted. The value of “False” means that alternative hypothesis was accepted. None of the proposed fitness functions have true location lower than 3227 man/h.

**Table 11:** The probability that fitness function calculate equation which is below the optimal standard UCP equation median

Fitness function	Probability (%)
MMRE	13
MdMRE	14
MEMRE	19
MdEMRE	10
MSE	23
RMSE	24
LAD	20
MAE	19
$R^2$	21
Pred(25)	9

Table 11 show the probability that fitness function calculate equation which is below the standard UCP equation median. As can be seen on this table, the best probability is provided by RMSE fitness function. The Pred(25) fitness function show the worst result only 9 equations from 100 equations are below 3227 man/h.

### Standard productivity factor

The standard productivity factor was set to 20. The median value for this productivity factor is 7469 man/h according to Fig. 5. The Wilcox signed rank test for one sample was used to determine which fitness function have a location shift lower than 7469 man/h. All calculation was performed on 95 % significance level.

**Table 12:** Hypothesis testing for standard productivity factor

Fitness function	p value	NULL hypothesis
MMRE	1	True
MdMRE	1	True
MEMRE	1	True
MdEMRE	1	True
MSE	1	True
RMSE	1	True
LAD	1	True
MAE	1	True
$R^2$	1	True
Pred(25)	1	True

Table 12 provides the results of Wilcox signed rank test for one sample. Every fitness function was tested on NULL hypothesis that this fitness function have lower true location than 7469 man/h. The value of “True” means that NULL hypothesis was accepted. The value of “False” means that alternative hypothesis was accepted. All fitness functions have true location lower than 7469 man/h.

**Table 13:** The probability that fitness function calculate equation which is below the standard UCP equation median

Fitness function	Probability (%)
MMRE	97
MdMRE	95
MEMRE	95
MdEMRE	81
MSE	97
RMSE	94
LAD	96
MAE	97
$R^2$	94
Pred(25)	86

Table 13 show the probability that fitness function calculate equation which is below the standard UCP equation median. As can be seen on this table, the best probability is provided by MSE, MAE and MMRE fitness functions. The MdMRE fitness function show the worst result 81 equations from 100 equations are below 7469 man/h.

## Threats to validity

It is widely recognised that several factors can bias the validity of empirical studies. Therefore, our results are not devoid of validity threats.

### External validity

External validity questions whether the results can be generalized outside the specifications of a study (Milicic and Wohlin 2004). Specific measures were taken to support external validity; for example, a random sampling technique was used to draw samples from the population in order to conduct experiments. Likewise, the statistical tests used in this paper, they are also quite standard. We note that the Wilcoxon method used in this paper features prominently. We used a relatively small size dataset, which could be a significant threat to external validity. Also the employed dataset contains projects related to one context that might be characterised by some specific properties. Similarly, we do not see how a smaller or larger dataset size should yield reliable results. It is widely recognised that, SEE datasets are neither easy to find nor easy to collect. This represents an important external validity threat that can be mitigated only replicating the study on another datasets. Another validity issue to mention is that either analytical programming nor differential evolution has been exhausted via fine-tuning. Therefore, future work is required to exhaust all the parameters of these methods to use their best versions. Threat to external validity could be also the implementation of the analytical programming and differential evolution algorithms. Although we used standard implementations, there is considerable amount of code, which could be the threat to validity.

### Internal validity

Internal validity questions to what extent the cause-effect relationship between dependent and independent variables hold (Alpaydın 2014). This paper used random sampling technique to assess methods. An alternate experimental condition would be to use N-way cross-validation. In theory, not using cross-validation is a threat to the validity of our results since we did not check if our results were stable across both random sampling technique and cross-validation.

## Discussion

The study started out with a goals of answering the overall research questions (RQ-1) of whether analytical programming technique outperformed the standard UCP equation. This question is answered in the result section. If the UCP method is optimized, via calibrating weight or via production factor, the analytical programming method is not efficient enough to outperform standard UCP equation. The evidence can be seen in result section in Table 10. As can be seen in this table, there is no fitness function with less median value then the standard UCP equation has on the significance level 95 %. On the other hand, if the productivity factor and the whole UCP is set to default value, there is a possibility, that model built by analytical programming outperform the standard UCP equation with any of proposed fitness functions.

There is also a another question (RQ-2), which must be answered. The results for answering this question is not as conclusive as we wanted to. For answering this question we need to study Tables 9, 11 and 13 from result section very carefully. From Table 9, can be seen that, MSE have the lowest median value as well as mean value and 3rd. quartile from the all of fitness functions. The maximum values, which can be seen in this table are caused by penalisation process of the evolution. With this in mind, we used median for comparison between fitness functions. The overall worst measurement result, measured by the median (MdMRE, MdEMRE), could be in its sensitivity to extreme values. The median is considered as an insensitive measure of centrality on data containing extreme values. Therefore, these measurements could be less suitable for the fitness functions. As can be seen in Tables 11 and 13, the MSE have a higher probability, that this fitness function built a model, which outperformed the standard UCP equation. If the standard productivity factor is used there is a 97 % probability, that MSE built a model more accurate then standard UCP equation. If the productivity factor is optimized there is a 23 % probability that MSE fitness function built a model, which outperformed standard UCP equation. The minimum from the whole study was calculated by MMRE fitness function. Nevertheless this minimum value was marked as a outlier as can be seen in Fig. 4.

## Conclusion

The current study found that the prediction accuracy measurement, which measures the median, performs worse than those that measure the mean or total value. Surprisingly, the MMRE measurement, which has raised a lot of controversy in the effort estimation field, could be considered as an average suitable fitness function. The results also revealed that fitness functions have a reasonably influence on the calculated predictions. Analytical programming method can be seen as a viable method for effort estimation. However, this is true if and only if the UCP method is not optimized. The MSE fitness function could be seen as the best fitness function due to her statistical properties. The findings of this study have a number of important implications for future research of the using of analytical programming as an effort estimation technique. More research is required to determine the efficiency of analytical programming for this task. It would be interesting to compare Karner’s model with one of the model built by analytical programming.
